# Deletion of *Mex3c* gene leads to autistic-like behavior in mice by inhibiting AMPK signal pathway

**DOI:** 10.3389/fnbeh.2025.1551440

**Published:** 2025-05-20

**Authors:** Hui Cai, Chengping Zhang, Haonan Zhang, Yong Du, Kai Wang

**Affiliations:** ^1^First School of Clinical Medicine, Ningxia Medical University, Yinchuan, China; ^2^Department of Pediatric Surgery, General Hospital of Ningxia Medical University, Yinchuan, China

**Keywords:** autism spectrum disorder, mitochondrial function, *Mex3c*, synaptic plasticity, social behavior, neurodevelopment

## Abstract

**Introduction:**

Autism Spectrum Disorder (ASD) is a hereditary neurodevelopmental condition influenced by genetic alterations, particularly in genes regulating neural development and synaptic plasticity. Emerging evidence suggests that the *Mex3c* gene plays a role in energy metabolism and neuronal development, indicating its potential relevance to ASD pathogenesis.

**Methods:**

To investigate the role of *Mex3c* in ASD, we generated *Mex3c* knockout (KO) mice and conducted a series of behavioral tests, histological analyses, and molecular assays. Behavioral phenotyping included elevated plus maze, open-field test, and three-chamber social interaction test. Histological assessments included Nissl staining, Golgi-Cox staining, and transmission electron microscopy. Molecular evaluations included Western blotting and analysis of the AMPK/SIRT1/PGC1α signaling pathway.

**Results:**

*Mex3c* KO mice exhibited autistic-like behaviors, including social deficits and anxiety-like traits. These behavioral abnormalities were accompanied by reduced neuronal number, decreased dendritic spine density, and impaired synaptic protein expression in the hippocampus. Mitochondrial structural damage and dysfunction were observed, alongside suppression of the AMPK/SIRT1/PGC1α signaling pathway.

**Conclusion:**

Our findings suggest that *Mex3c* gene deletion induces ASD-like phenotypes in mice, potentially through disruption of mitochondrial function and synaptic integrity via the AMPK/SIRT1/PGC1? pathway. These results support the candidacy of *Mex3c* as a susceptibility gene for ASD and highlight mitochondrial signaling pathways as potential therapeutic targets.

## 1 Introduction

Autism Spectrum Disorder (ASD) is characterized as a heritable neurodevelopmental disorder associated with social challenges in terms of communication and interaction, along with restricted and repetitive behavioral changing patterns (Thabtah, 2019; Takumi et al., 2020). Various neurological complications such as epilepsy, cerebral palsy, macrocephaly, hydrocephalus, and congenital anomalies are associated with these disorders, as revealed by the long-term follow-ups of clinical autism patients (Pan et al., 2021). The etiological aspects of ASD, however, remain unclear, but the identified potential contributors include parental psychiatric disorders, genetic factors, and psychotropic drugs fetal exposure (Genovese and Butler, 2020). Recent investigations have demonstrated genetic factors, specifically deletion or mutation of a certain gene, as the major causes of ASD (Courchesne and Pierce, 2005). Any alteration in ASD risk genes affects the downstream signaling pathways and leads to their dysregulation, resulting in abnormal cellular proliferation, differentiation, and synaptic plasticity of neuronal cells, ultimately triggering the onset of ASD (Willsey et al., 2013; Stoner et al., 2014).

MEX-3 is a highly conserved RNA-binding protein from Caenorhabditis elegans to humans. It serves as a crucial player in various physiological phenomena, including metabolic processes, hypertension, growth/proliferation, and immune regulation. The four human and mouse homologs of the *MEX-3* gene (*MEX3A*, *MEX3B*, *MEX3C*, and *MEX3D*) each consist of two K homology (KH) RNA-binding domains at their N-terminus along with a zinc finger domain at the C-terminus to mediate protein-protein interactions (Draper et al., 1996; Buchet-Poyau et al., 2007). Various studies have investigated that *Mex3c* gene knockout leads to significant negative energy balance (Han et al., 2014), and its KH domain is responsible for regulating early aspects of embryonic developmental stages (Hollingworth et al., 2012). Our preliminary research has demonstrated that *Mex3c* gene inactivation in mice causes both neurodevelopmental deficiencies and an increased probability of cognitive and nervous disorders (Du et al., 2020; Wang et al., 2023), suggesting *Mex3c* as a possible new risk gene for ASD.

Oxidative stress and functional impairment are strongly associated with the development of ASD, as suggested by increasing literature evidence. Structural and functional damage to brain and neuronal cells is initiated due to the accumulation of damaged mitochondria. Gandal et al. (2018) found that autism and schizophrenia are strongly characterized by significant downregulation of mitochondria-related genes. Marui et al. (2011) reported a genetic variation, i.e., single nucleotide polymorphism (SNP), in the mitochondrial gene (*NDUFA5*) associated with autism. Additionally, mitochondrial function is also found to be altered in ASD. Reduction in the expression of specific ETC complex has been shown in certain brain regions of ASD children, as revealed by many studies, demonstrating that ASD arises from mitochondrial dysfunction rather than the primary mitochondrial disease (Chauhan et al., 2011).

AMP-activated protein kinase (AMPK), a serine/threonine protein kinase, offers a fundamental role in the energy homeostasis of cells and is crucial for the regulation of mitochondrial activities (Jeon, 2016; Chiang et al., 2018). AMPK serves as the primary energy sensor, maintains cellular energy dynamics, and regulates cell cycle and oxidative stress-induced apoptosis (Chiang et al., 2016; Jeon, 2016; Chiang et al., 2018). Studies suggest that over-activation or suppression of AMPK is associated with various signaling mechanisms toward the progression of neurodegenerative pathologies (Garcia and Shaw, 2017; Lin and Hardie, 2018). A member of the Sirtuin protein family (SIRT1), it is involved in aging, transcription, metabolic processes, and mitochondrial biogenesis and is found to be regulated by AMPK (Lempiainen et al., 2013; Tian et al., 2019). SIRT1 integrates the level of NAD+ and activates PGC1a through deacetylation to promote mitochondrial biogenesis (Rodgers et al., 2005). Although the *Mex3c* gene influences mitochondrial damage through the AMPK/SIRT1/PGC1a signaling pathway, the mechanism remains unclear.

In the current research, we developed the *Mex3c* KO model to investigate the potential role of *Mex3c* and its specific mechanism for the development of ASD. We found that *Mex3c* gene-deficient mice exhibited significant social and behavioral disorders. Evidence from the proteomic analysis of the hippocampus revealed the *Mex3c* gene as the potential regulator of the AMPK/SIRT1/PGC1a signaling pathway. The absence of this gene results in an increased expression of AMPK protein, subsequent decline in SIRT1 protein, and elevated acetylation of PGC1a.

These findings demonstrated that loss of the *Mex3c* gene is responsible for disrupting neuronal mitochondrial function through aberrant activation of the AMPK/SIRT1/PGC1a signaling pathway, thereby contributing to the ASD onset.

## 2 Materials and methods

### 2.1 Animals

We selected about 7 weeks-old mice for the experiment. *Mex3c+/-* gene knockout mice were obtained based on C57BL/6J mice (procured from Saiye Experimental Animal Technology Co., Ltd.) [SCXK (Guangdong) 20180032]. *Mex3c+/-* mice were propagated, and the genotypes of their offspring were identified by PCR assay, and *Mex3c+/+* and *Mex3c-/-* were obtained. All the experimental animals were kept in a sterile environ-ment; 30 *Mex3c+/+* and 30 *Mex3c-/-* mice were used for a light-dark cycle at an interval of 12 h, and the room temperature was maintained at 25°C. The mice had free access to eat and drink throughout the study duration. The Medical Research Ethics Review Committee of the General Hospital of Ningxia Medical University approved all the experimental protocols of the study (Approval Number: KYU-20220028).

### 2.2 Behavioral assessment

#### 2.2.1 Open field test

The mice were housed in the center of a white open blank space (long, 50 cm × wide, 50 cm × high, 30 cm) and were allowed to explore freely for 5 min. During this duration, the trajectory of the mice while doing exercise was recorded; after each experimentation, the experimental site was thoroughly cleaned and disinfected with 75% ethanol. The anxiety-like behavior of the mice was evaluated by comparing the stay time in the central area (25 cm long by 25 cm wide).

#### 2.2.2 Elevated cross maze

The experimental apparatus of the elevated cross maze comprised two open and two closed arms, which are perpendicular to each other (50 cm above the ground and 20 cm high on the wall). The mice were placed in the central area of the EPM (10 × 10 cm), facing the open arm, and they were allowed to explore the maze freely for 5 min. A digital camera was used for recording, and SAMRT3.0 software was used for the behavioral analysis. The time and entry times of mice in open and closed arms were recorded. Anxiety-like behavior was determined by calculating the percentage of open-arm stay time to total time (open-arm residence time + closed-arm residence time).

#### 2.2.3 Three boxes of social experiments

The three-chamber test was conducted to examine social behavior and social novelty in mice. The experimental apparatus was a rectangular box (123 cm × 50 cm × 40 cm) divided into three equal chambers by transparent partitions with openings that allowed free movement between compartments. A cylindrical wire cage (rat cage) was placed in the left and right side chambers to hold stimulus mice. The test consists of two stages: Stage 1 is the social approach test; the test mouse was placed in the center chamber and allowed to explore all three chambers freely for 5 min. One side chamber contained an unfamiliar mouse enclosed in a wire cage, while the other chamber contained an empty cage. The time spent interacting with the unfamiliar mouse versus the empty cage was recorded. Stage 2 is the social novelty test; a new, unfamiliar mouse was placed in the previously empty cage. The test mouse was again allowed to explore for 5 min. The time spent interacting with the familiar versus the novel mouse was measured to evaluate social memory and novelty preference. The social preference index (SPI) was calculated using the following formula: SPI = (time spent with the stranger mouse–time spent in the empty chamber)/(time spent with the stranger mouse + time spent in the empty chamber). All sessions were recorded, and social interaction time and preference indices were analyzed using the Smart3.0 behavioral analysis system.

### 2.3 Tissue processing

Once the behavioral analysis was done, isoflurane was used to anesthetize the mouse. The whole brain was harvested and instantly processed for fixation in a neutral tissue fixation solution (Wuhan Seville Biotechnology Co., Ltd., China) for 12 h at 4°C. Brain tissue was transferred to 70% alcohol solution and allowed for dehydration for 24 h, followed by embedding in paraffin as per the standard protocol. The embedded blocks were cooled and stored at 20°C. A Paraffin slicer was used to cut 3-micron slices and taken on glass slides for further experiments.

### 2.4 Western blot analysis

Total protein content from the hippocampus of mice was extracted using the extraction kit (Jiangsu Kaiji Biotechnology Co., Ltd.) and quantified via the BCA protein concentration kit (Jiangsu Kaiji Biotechnology Co., Ltd.). The same amount of protein (40 μg per reagent tube) was electrophoresed on 10 % SDS-PAGE and transferred on a 0.45 μm PVDF membrane. The membrane was incubated with protein-blocking solution for 20 min (Wuhan Seville Biotechnology Co., Ltd., China), followed by incubation with antibodies (GAP-43,PSD-95,pAMPK, AMPK,SIRT1,PGC1a,GAPDH)at 4°C for 16 h, PSD95 (ab18258, Abcam; dilution, 1:1000);GAP-43 (ab75810,Abcam;dilution,1:1000); AMPK (GB114323, Servicebio;dilution, 1:1000); pAMPK (GB121919, Servicebio; dilution, 1:1000); SIRT1 (GB11512, Servicebio; dilution, 1:1000); PGC1a (GB11912, Servicebio; dilution, 1:1000); GAPDH (GB15002, Servicebio; dilution, 1:5000); the membrane was washed with Tris buffer saline (TBS) containing 5% Tween20,and the secondary antibody Alexa Fluor 790 (ab186695, Abcam; dilution 1:10000) and IRDye 680RD (ab216777, Abcam; dilution 1:10000) were incubated for 1 h at RT (25°C), Antibody Dilution Buffer was used to dilute antibodies (Shanghai Ya enzyme Biopharmaceutical Technology Co., Ltd.) using the optimal dilution ratio of each antibody. Protein expression was quantified via ImageJ.

### 2.5 Nissl staining

The paraffin sections were dewaxed with xylene for 5–10 min. Then fresh xylene was used to dewax again for 5–10 min. Sections were dehydrated using a descending series of anhydrous ethanol (90% for 5 min, 80% for 3 min, 70% for 3 min). The sections were washed gently with water for 2 min. Nissl’s dye solution (China Wuhan Seville Bio-technology Co., Ltd.) was added to stain the slices for 5 min. Finally, the neutral resin film was used to scan and capture pictures, and ImageJ was used for quantitative analysis. Coronal brain sections containing the hippocampus were obtained from Bregma -1.67 mm, based on the Paxinos and Franklin Mouse Brain Atlas. For quantification of Nissl-positive cells, stained brain sections encompassing the hippocampal regions [CA1, CA3, and dentate gyrus (DG)] were imaged under a light microscope at 40 × magnification. In each region of interest, we randomly selected three non-overlapping fields of view per section from each animal. The number of Nissl-stained neurons was manually counted. To account for variations in cell density, the total number of Nissl-positive cells was normalized to the total number of cells (including both Nissl-positive and Nissl-negative cells) within the same field of view. All counts were performed by two independent, blinded observers to minimize bias.

### 2.6 IHC

Coronal brain sections containing the hippocampus were obtained from Bregma -1.67 mm, based on the Paxinos and Franklin Mouse Brain Atlas. The paraffin-embedded tissue sections were dewaxed with xylene for 5–10 min. Then fresh xylene was used to dewax again for 5–10 min. Section dehydration was done using anhydrous ethanol for 5 min, fresh anhydrous ethanol 5 min, then 90% ethanol treatment, 80% ethanol treatment, 3 min ethanol treatment, 70% ethanol treatment, 3 min, and water washing for 2 min. NeuN/Ki67 antibody was used for immunohistochemical analysis. The first antibody incubation proceeded for 24 h. Goat anti-mouse IgG antibody was incubated for 30 min, followed by incubation in DAB chromogenic agent for 5–10 min. The slices were dipped in hematoxylin stain (Wuhan Seville Biotechnology Co., Ltd., China) for 5 min. Neutral resin film was used to scan the sections and to capture images. Images of the hippocampal CA1, CA3, DG regions were captured under a microscope at consistent 100 × magnification. NeuN expression was performed on hippocampal tissue sections. Three randomly selected, non-overlapping fields were analyzed within each of the CA1, CA3, and DG subregions of the hippocampus. NeuN expression was quantified using ImageJ software, specifically by measuring the integrated density (IntDen) value, which represents the product of area and mean pixel intensity. To normalize NeuN expression, IntDen values were divided by the total selected area, yielding a relative expression value.

### 2.7 Transmission electron microscopy (TEM)

Transmission electron microscopy (TEM) images of hippocampal neurons were analyzed to assess mitochondrial integrity. Samples were dehydrated using a graded ethanol series (50%, 70%, 90%, and 100%) and 100% acetone, each for 15 min, followed by 4 h embedding. Ultrathin sections (70 nm) were prepared and mounted on copper grids. The sections were stained with uranyl acetate and lead citrate. Mitochondrial morphology and abundance were observed under a transmission electron microscope at ≥ 50,000 × magnification. For quantitative analysis, 16 cells from four randomly selected fields of view were assessed to determine mitochondrial counts, and statistical analyses were performed. Mitochondria were categorized as damaged based on the presence of characteristic pathological features as previous described (Lam et al., 2021), including mitochondrial swelling, cristae loss or fragmentation, vacuolization, and outer membrane rupture. The percentage of damaged mitochondria was calculated as the number of damaged mitochondria divided by the total number of mitochondria observed within the selected neurons.

### 2.8 Mitochondrial membrane potential analysis (ΔΨm) and JC-1 flow cytometry

Mitochondrial membrane potential was evaluated using the JC-1 assay Kit (Beijing Solarbio Science and Technology Co., Ltd). Cells were incubated with 5 μM JC-1 dye at 37°C for 20 min. CCCP (10 μM) was used as a positive control to induce mitochondrial depolarization. After incubation, cells were washed twice with cold PBS and centrifuged at 400 × *g* for 5 min each time to remove residual dye. Cells were then gently resuspended in 500 μL of PBS, transferred into standard flow cytometry tubes, and immediately analyzed using a BD FACSCanto II flow cytometer (BD Biosciences). At least 10,000 events per sample were collected for analysis. The fluorescence intensities of JC-1 monomers (green, 530 nm) and aggregates (red, 585 nm) were recorded, and the red-to-green fluorescence ratio was calculated as an index of mitochondrial membrane potential. A reduced red/green fluorescence ratio indicated mitochondrial depolarization.

### 2.9 Golgi-cox staining

Three mice each from the KO and WT groups were selected and anesthetized via intraperitoneal injection of sodium pentobarbital. After euthanasia, the mice were decapitated on ice, and the entire brain was rapidly extracted. Using a blade, coronal tissue blocks of the hippocampal region, approximately 5–10 mm thick, were carefully dissected. Coronal tissue blocks were prepared from the same bregma range -1.79 mm. The tissue blocks were briefly rinsed with distilled water and then immersed in Golgi staining fixative for approximately 5 days. Subsequently, the tissue blocks were sectioned into 50 μm thick slices. The slices were dehydrated twice in absolute ethanol for 20 min each, followed by clearing in xylene for 30 min. Finally, the sections were scanned. Dendritic spine was analyzed using Golgi-Cox staining. Images were captured under an optical microscope at 20 × and 100 × objective magnification. For each region, three neurons per mouse were randomly selected, and three dendritic segments per neuron were analyzed. Dendritic spine density was quantified by manually counting the number of spines along the length of each dendritic segment using ImageJ software, and the density was expressed as number of spines per 20 μm of dendrite length.

### 2.10 Proteomic sample preparation and mass spectrometry analysis

#### 2.10.1 Protein extraction and quantification

Brain tissues were homogenized in 300 μL of lysis buffer containing 8 M urea and protease inhibitors (50:1 ratio). Samples were subjected to ultrasonication on ice (1 s on/2 s off, total 120 s), followed by centrifugation at 14,000 × *g* for 20 min at 4°C. The supernatant was collected for protein quantification using the Bradford assay. Protein concentration was determined by measuring absorbance at 595 nm after incubation with Coomassie Brilliant Blue G250. Standard curves were generated using bovine serum albumin (BSA) standards.

#### 2.10.2 Protein digestion and desalting

A total of 100 μg of protein from each sample was reduced with 10 mM DTT at 37°C for 1 h and alkylated with 40 mM iodoacetamide (IAM) at room temperature in the dark for 45 min. The samples were diluted with 50 mM ammonium bicarbonate (pH 8.0), and digested overnight at 37°C with trypsin (enzyme-to-substrate ratio 1:50). The digestion was terminated with 0.1% formic acid (FA), and peptides were desalted using C18 columns. Columns were activated with 100% acetonitrile (ACN), equilibrated with 0.1% FA, and peptides were eluted using 70% ACN. Eluates were dried via vacuum centrifugation.

#### 2.10.3 LC-MS/MS analysis

Dried peptides were reconstituted in 10 μL of mobile phase A (0.1% FA in water), centrifuged at 14,000 × *g* for 20 min at 4°C, and 1 μg of peptide was injected per sample. Peptide separation was performed using a nanoLC system (RIGOL L-3000) coupled to a Q Exactive HF-X mass spectrometer (Thermo Fisher Scientific) equipped with a Nanospray Flex™ ion source. The LC gradient consisted of increasing buffer B (80% ACN, 0.1% FA) from 8% to 95% over 80 min at a flow rate of 600 nL/min.

The mass spectrometer operated in data-dependent acquisition (DDA) mode. Full MS scans were acquired over an m/z range of 350–1,550 with a resolution of 120,000 (at m/z 200), AGC target of 3 × 10^6^, and maximum injection time of 80 ms. The top 40 most intense precursor ions were fragmented via higher-energy collision dissociation (HCD) at 27% normalized collision energy and analyzed in MS/MS scans at a resolution of 15,000, AGC target of 5 × 10^4^, and maximum injection time of 45 ms. Raw MS files were analyzed using Proteome Discoverer software (version 2.4; Thermo Fisher Scientific). Spectra were searched against a UniProt mouse protein database.

### 2.11 Statistical analysis

All statistical analyses were performed using GraphPad Prism (version 10.1.2) and R software (version 4.2.0). Data are expressed as mean ± standard deviation (SD). All summary statistical data are presented in Supplementary Table 1. For comparisons between two groups, unpaired two-tailed *t*-tests were used. For multiple group comparisons, two-way ANOVA followed by Bonferroni’s *post hoc* test was applied where appropriate. The *P*-value < 0.05 was considered statistically significant. Significance levels are indicated as follows: *P < 0.05; **P < 0.01; ***P < 0.001; ****P < 0.0001.

## 3 Results

### 3.1 *Mex3c* gene deletion results in autistic-like behavior in mice

This study aimed to investigate whether *Mex3c*-deficient mice exhibit an increased tendency toward autism-like behaviors. We used *Mex3c+/-* mice were bred to obtain *Mex3c-/-* (KO) and *Mex3c+/+* (WT) genetically modified mice. A series of behavioral tests, including the three-chamber social interaction test, elevated plus maze, and open-field test, were conducted to assess autistic-like behaviors in the KO group.

**FIGURE 1:**
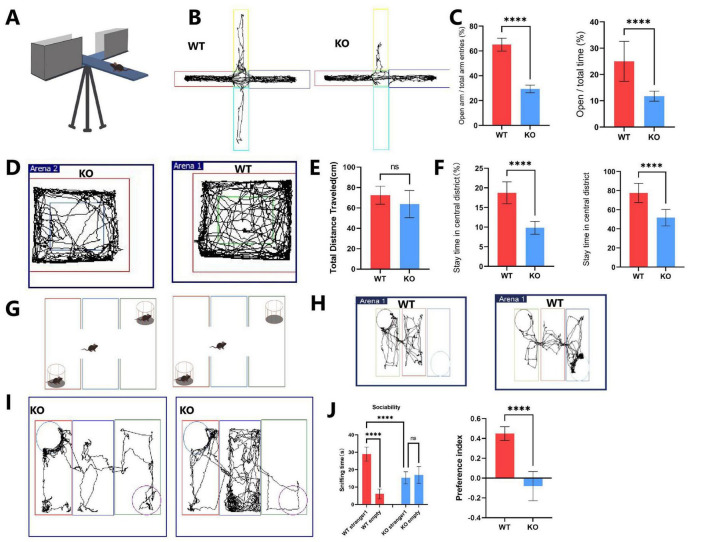
Behavioral tests evaluating autistic-like behaviors in *Mex3c* KO mice. **(A)** Representative image of the elevated plus maze. **(B)** Locomotor activity trace of WT and KO mice in the elevated plus maze. **(C)** Percentage of entries into the open arms (left) and percentage of time spent in the open arms (right). **(D)** Locomotor activity traces of WT and KO mice in the open-field test. **(E)** Quantification of total distance traveled, **(F)** Percentage of time spent in the center zone (left), and number of entries into the center zone (right). **(G)** Representative image of the three-chamber social interaction test. **(H)** Locomotor activity trace of WT mice in stages 1 and 2 of the three-chamber test. **(I)** Locomotor activity trace of KO mice in stages 1 and 2 of the three-chamber test. **(J)** Comparison of time spent in each chamber during stage 1 (habituation) and stage 2 (social interaction) in WT and KO mice. **(K)** Quantification of the social preference index. Open field test: WT, *n* = 8; KO, *n* = 8. Elevated plus maze: WT, *n* = 16; KO, *n* = 16. Three-chamber test: WT, *n* = 6; KO, *n* = 6. Data are presented as mean ± SD. Statistical significance was determined using unpaired *t*-tests for two-group comparisons and two-way ANOVA for the chamber sniffing times (stranger mouse vs. empty chamber). ****P < 0.0001.

In the elevated plus maze, the KO group exhibited significantly increased anxiety-like behavior (Figures 1A, B). Specifically, the percentage of time spent in the open arms (OT = 11.75 ± 1.84%) and the percentage of entries into the open arms (OE = 29.35 ± 2.97%) were markedly lower in the KO group compared to the WT group (OT = 24.97 ± 7.39%, OE = 65.08 ± 5.10%) (Figure 1C). In the open field test, although the total distance traveled did not differ significantly between the WT and *Mex3c* KO mice, *Mex3c* KO mice spent significantly less time in the center area compared to WT controls (Figures 1D, E), indicating elevated anxiety-like behavior. The number of entries into the center zone and time per entry were also reduced in the KO group (*P* < 0.05) (Figures 1F). These findings suggest that the decreased center exploration reflects increased anxiety rather than hypoactivity or general locomotor impairment.

In the three-chamber social interaction test, WT mice spent significantly more time interacting with the stranger mouse compared to the empty chamber (*P* < 0.01), indicating normal social preference behavior (Figures 1G–J). In contrast, *Mex3c* KO mice did not show a significant difference in time spent between the stranger mouse and the empty chamber (*P* > 0.05), indicating impaired social preference (Figure 1J). Moreover, when comparing groups, *Mex3c* KO mice spent significantly less time with the stranger mouse compared to WT mice (*P* < 0.05) (Figure 1J). Additionally, the social preference index was significantly lower in the KO group (*P* < 0.05), a positive SPI indicates a preference for social interaction, further confirming impaired sociability (Figure 1K).

### 3.2 *Mex3c* gene deletion reduces the number and maturity of Nissl bodies in hippocampal neurons of mice

**FIGURE 2:**
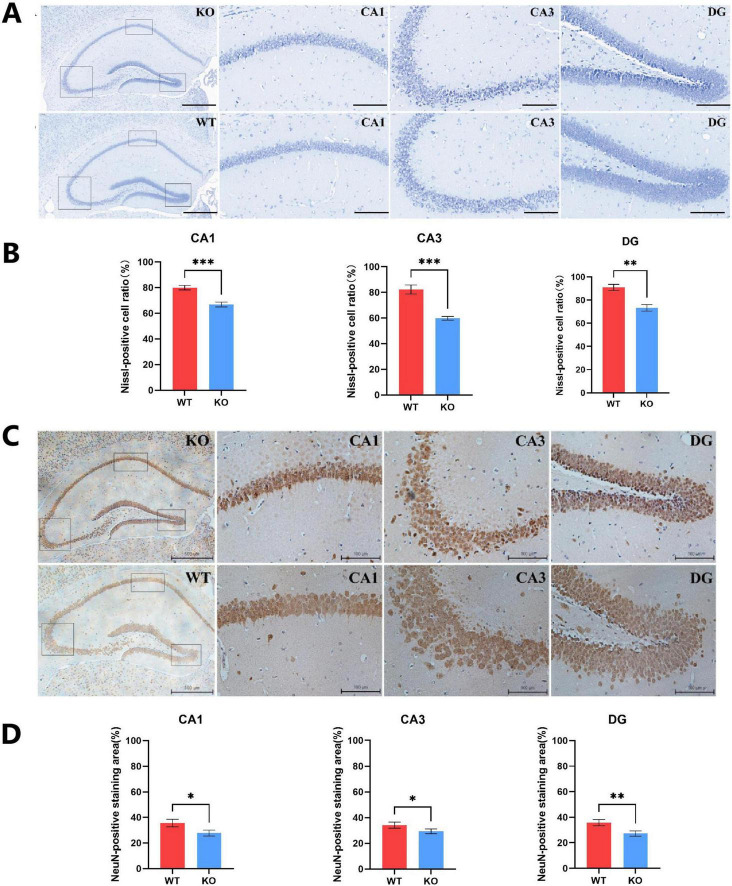
Hippocampal neuronal integrity in *Mex3c* KO mice. **(A)** Representative images of hippocampal Nissl staining in WT and KO mice (scale bar: 100 μm). The black boxes indicate the CA1, CA3, and DG regions in the hippocampus of KO mice (magnified images, scale bar: 20 μm). **(B)** Quantification of Nissl-positive cells in the CA1, CA3, and DG regions of the hippocampus. **(C)** Representative images of hippocampal NeuN immunohistochemical staining in WT and KO mice (scale bar: 500 μm). The black boxes correspond to the CA1, CA3, and DG regions of KO mice (magnified images, scale bar: 100 μm). **(D)** Quantification of NeuN-positive cells in the CA1, CA3, and DG regions of the hippocampus. Data are presented as mean ± SD. Statistical significance was determined using unpaired *t*-tests. WT, *n* = 6; KO, *n* = 6. *P < 0.05; **P < 0.01; ***P < 0.001.

Behavioral analysis showed that deletion of *Mex3c* increased autism-like and repetitive behaviors in mice. This is consistent with the previous experiments reported by our group (Du et al., 2020; Wang et al., 2023). We also examined the structural alterations in the mouse brain due to *Mex3c* gene knockout. Nissl staining was performed to assess neuronal damage in both WT and KO mice (Kern and Jones, 2006; Ebert and Greenberg, 2013). The results showed that Nissl bodies in the CA1 and CA3 regions of the hippocampus in WT mice appeared dark blue, numerous, and well-organized. However, KO mice indicated lighter staining, distorted arrangement, and a reduced number of Nissl bodies with distorted boundaries (Figure 2A). To comprehensively assess the impact of *Mex3c* deficiency on neuronal integrity, we performed Nissl staining and quantified the ratio of Nissl-positive cells to total cells in CA1, CA3, and dentate gyrus (DG) of the hippocampus. Quantitative analysis revealed *Mex3c* KO mice exhibited markedly lower Nissl-positive cell ratios in the CA1 (*P* < 0.01), CA3 (*P* < 0.01), and DG (*P* < 0.05) regions relative to WT mice (Figure 2B). These findings indicate widespread neuronal impairment in hippocampal areas in *Mex3c* KO mice, suggesting that *Mex3c* deficiency compromises neuronal maintenance in multiple brain regions critical for cognition and social behavior. Furthermore, the maturity of neuronal cells was also assessed. IHC staining revealed a lower proportion of NEUN-positive cells in the KO group in comparison to the WT group (Figures 2C, D).

### 3.3 *Mex3c* gene deletion resulted in reduced synaptic phenotypes of mice’s cerebral cortex and hippocampus

**FIGURE 3:**
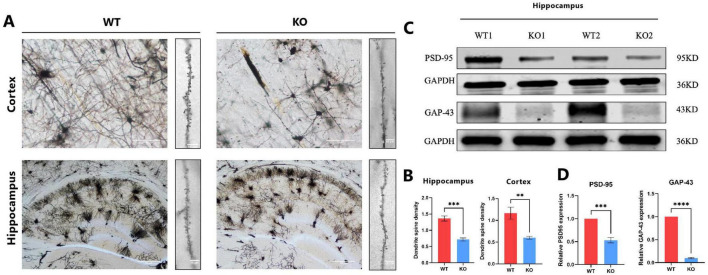
Synaptic structural and protein alterations in *Mex3c* KO mice. **(A)** Representative images of pyramidal neuron dendritic morphology in the cortical and hippocampal regions of WT and KO mice, Scale bars: 50 μm (low magnification) and 10 μm (high magnification) for cortex images; 200 μm (low magnification) and 10 μm (high magnification) for hippocampus images. **(B)** Quantification of the dendritic spine density. **(C)** Representative western blot images showing the expression of synaptic proteins PSD-95 and GAP-43 in the hippocampus. Representative samples from two independent animals per group are shown (WT1, WT2, KO1, KO2). **(D)** Quantification of PSD-95 and GAP-43 protein expression levels. Data are presented as mean ± SD. Statistical significance was determined using unpaired *t*-tests. WT, *n* = 6; KO, *n* = 6. ***P < 0.001; **P < 0.01; ****P < 0.0001.

Dendritic spines are essential structural components of excitatory synapses in the central nervous system (CNS), and their density and morphology are critical determinants of synaptic connectivity and plasticity (Ultanir et al., 2007). To investigate whether *Mex3c* deficiency affects synaptic structure, we quantitatively analyzed dendritic spine density in pyramidal neurons of the cerebral cortex and hippocampus. Secondary dendritic branches approximately 20 μm in length were selected for spine quantification. The results revealed a significant reduction in dendritic spine density in *Mex3c* KO mice compared to WT controls (Figures 3A, B), suggesting impaired synaptic structure in the absence of *Mex3c* (Figure 3A). Furthermore, the quantification result revealed a significant reduction in dendritic spine density in *Mex3c* KO mice compared to WT controls (Figure 3B). This suggests impaired synaptic plasticity associated with *Mex3c* deficiency. Consistent with these structural abnormalities, Western blot analysis of synaptic-associated proteins in the hippocampus showed reduced expression of PSD-95 and GAP-43 in *Mex3c* KO mice compared to WT controls (Figures 3C, D). Together, these findings indicate that *Mex3c* deletion impairs synaptic integrity and plasticity, which may underlie the neurodevelopmental deficits observed in *Mex3c* KO mice.

### 3.4 Mitochondrial abnormalities in hippocampal neurons caused by *Mex3c* gene deletion

**FIGURE 4:**
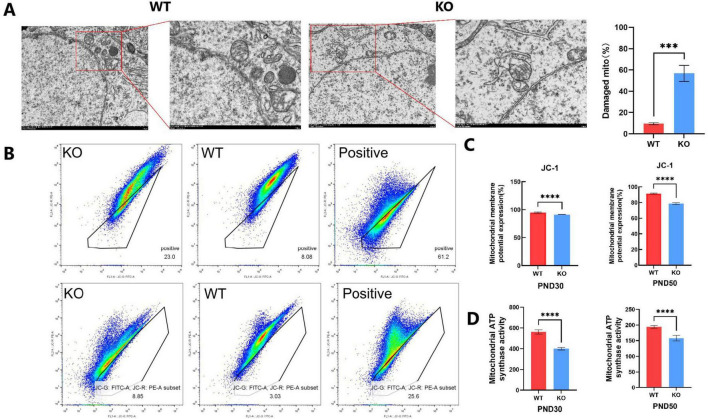
Mitochondrial structural and functional abnormalities in *Mex3c* KO mice. **(A)** Representative transmission electron microscopy images of hippocampal neurons in WT and KO mice (right: cell morphology at 8,000 × magnification, scale bar = 2 μm; mitochondrial ultrastructure at 15,000 × magnification, scale bar = 1 μm). Quantification of mitochondrial damage in hippocampal neurons is shown on the right; the percentage of damaged mitochondria was calculated relative to the total mitochondria analyzed. **(B)** Flow cytometry analysis of mitochondrial membrane potential using JC-1 staining in postnatal day 30 (PND30) and postnatal day 50 (PND50) mice. CCCP (10 μM) was used as a positive control to induce mitochondrial depolarization (right). **(C)** Quantification of mitochondrial membrane potential based on JC-1 assay results. **(D)** Quantification of mitochondrial ATP synthase activity in PND30 and PND50 mice. Data are presented as mean ± SD. Statistical significance was determined using unpaired *t*-tests. WT, *n* = 6; KO, *n* = 6. ***P < 0.001; ****P < 0.0001.

Transmission electron microscope was used to examine the hippocampal ultrastructure during brain development in mice to evaluate the effect of *Mex3c* gene deletion. In the WT group, mitochondria displayed a normal oval shape with well-defined cristae. In contrast, mitochondria in the *Mex3c* KO group exhibited blurred membranes, partial structural damage, varying degrees of swelling, numerous vacuoles, and poorly defined or absent cristae, along with overall enlargement, deformation, and marked structural abnormalities (Figure 4A). Quantitative analysis showed that the percentage of damaged mitochondria was significantly higher in *Mex3c* KO mice compared to WT controls (*P* < 0.01) (Figure 4A). The damage of mitochondria is hypothesized to be a significant factor underlying autism spectrum disorder in mice resulting from the knockout of the *Mex3c* gene.

Following the identification of the atypical arrangement of mitochondria in the mouse hippocampus using a transmission electron microscope, we proceeded to measure the mitochondrial membrane potential. Mitochondrial membrane potential (Δψ m) is an important indicator of cell health and functional status, which is widely used to study the behavior of mitochondria-related to cell pathways. The most significant is apoptosis (Perelman et al., 2012). Furthermore, the study of mitochondrial membrane potential is an important aspect for the evaluation of mitochondrial function or activities because it reveals the difference of potential and represents the crucial components of the proton electrochemical gradient responsible for the production of more than 90% of the total available respiratory energy (Palmeira and Rolo, 2012). We divided the mice into two groups based on their age: one group was 30 days old, and the other group was 50 days old. The mitochondrial membrane potential was measured using JC-1 mitochondrial membrane potential detection kit followed by flow cytometry. The results of JC-1 detection of 30 days-old and 50 days-old mice showed that there were cells with loss of mitochondrial membrane potential in the black frame and cells outside the region that might still have mitochondrial membrane potential. The mitochondrial membrane potential of WT and KO cells was displayed in the middle and left images, respectively (Figure 4B). The number of cells with loss of mitochondrial membrane potential in KO is higher than that in WT, and the positive control (Figure 4B). The findings demonstrated a substantial decrease in the number of mitochondria with membrane potential in the KO group compared to the WT group in both groups of mice. Among them, the selected region was the inactivated cell mass of mitochondria (JC-1 was detected), and the outer region was the normal cell mass of mitochondrial membrane potential (no JC-1 was detected) (Figure 4B). In comparison to the WT group, the number of mitochondrial membrane potential loss cells in the KO group increased significantly. The results indicated that the function of mitochondrial energy metabolism was inhibited due to the deletion of the *Mex3c* gene. At the same time, the changes in mitochondrial membrane potential in mice once again proved the increase of neuronal apoptosis in the hippocampus, which was consistent with the previous results of western blot detection (Figure 4C). We analyzed the ATP synthase activity of the mitochondrial respiratory chain. The findings revealed that the ATP synthase activity in the KO group, where the *Mex3c* gene was deleted, was significantly lower compared to the WT group (Figure 4D). This indicates that the deletion of the *Mex3c* gene not only impacted the integrity of the mitochondrial structure but also hindered the production of ATP in the mitochondrial respiratory chain. Consequently, this led to abnormal cellular energy metabolism and had a subsequent impact on nerve development.

### 3.5 *Mex3c* gene deletion blocks the abnormal AMPK/SIRT1/PGC1a signaling pathway and leads to mitochondrial damage

**FIGURE 5:**
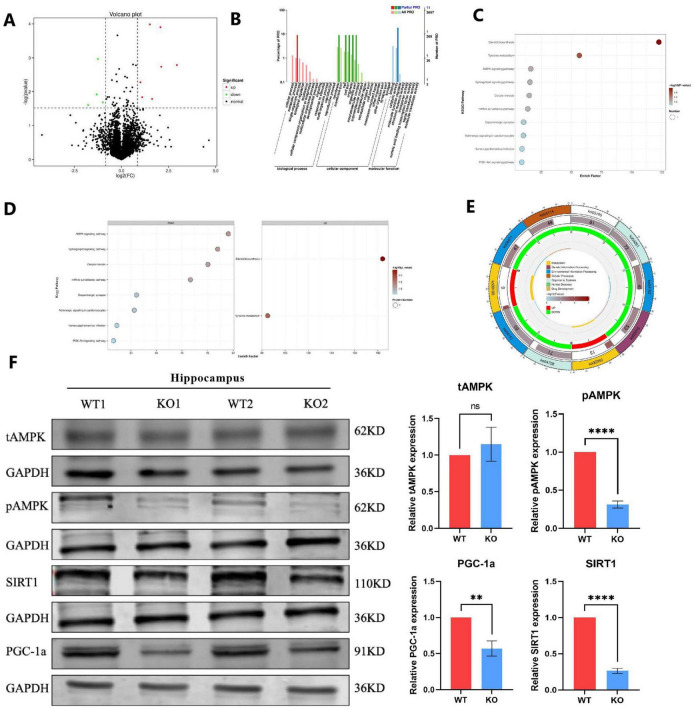
Proteomic and pathway analysis reveals dysregulation of the AMPK/SIRT1/PGC1a signaling pathway in *Mex3c* KO mice. **(A)** Volcano plot of differentially expressed proteins between WT and KO mice. Red dots represent upregulated proteins, while green dots represent downregulated proteins. **(B)** Gene Ontology (GO) enrichment analysis of differentially expressed proteins. **(C)** Overview of Kyoto Encyclopedia of Genes and Genomes (KEGG) pathway enrichment analysis. **(D)** Detailed KEGG enrichment results. The left side shows pathways enriched for downregulated proteins, while the right side shows pathways enriched for upregulated proteins. **(E)** KEGG pathway map highlighting affected metabolic cycles. **(F)** Western blot analysis of tAMPK, pAMPK, SIRT1, and PGC1a protein expression in the hippocampus. Representative samples from two independent animals per group are shown (WT1, WT2, KO1, KO2). Data are presented as mean ± SD. Statistical significance was determined using unpaired *t*-tests. WT, *n* = 6; KO, *n* = 6. **P < 0.01; ****P < 0.0001.

We employed proteomic and mass spectrometry analysis to examine the protein constituents of the brain tissues of *Mex3c* KO and WT mice to investigate the mechanism underlying the impact of *Mex3c* gene deletion on the mouse brain. Proteins with a fold change (FC) ≥ 1.80 and *P*-value ≤ 0.03 were considered upregulated, while proteins with FC ≤ 0.56 (inverse of 1.80) and *P*-value ≤ 0.03 were considered downregulated. Using these criteria, we identified a total of 11 differentially expressed proteins (DEPs), including seven downregulated and 4 upregulated proteins. The DEPs were visualized using a volcano plot, and the top 10 upregulated and downregulated proteins were highlighted (Figure 5A). Protein identities were confirmed through searches of the UniProt database.

To analyze the biological functions of these DEPs, we analyzed the GO function of DEPs from three levels: sub-function (molecular function, MF), biological process (biological process, BP), and fine cell component (cellular component, CC). Comparing the results to the WT group, it was found that MF primarily consisted of binding, catalytic activity, and molecular function regulator; BP included biological regulation, single organism processes, cellular and metabolic processes, localization, cellular component organization or biogenesis, multicellular organism processes, and developmental processes; CC included membrane, membrane part, cell, cell part, extracellular region, extracellular region part, membrane, extracellular region (Figure 5B). Subsequently, we conducted pathway enrichment analysis using the Kyoto Encyclopedia of Genes and Genomes (KEGG) database, applying a hypergeometric test to determine significantly enriched pathways among the DEPs (Figure 5C). Pathway significant enrichment can be used to identify the key metabolic and signal transduction pathways that are involved in differential proteins. KEGG enrichment results showed a total of 10 related signal pathways, including AMPK signaling pathway, Sphingolipid signaling pathway, Oocyte meiosis, mRNA surveillance pathway, Dopaminergic synapse, Adrenergic signaling in cardiomyocytes, Human papillomavirus infection and PI3K-Akt signaling pathway; up-regulated pathways, including Steroid biosynthesis and Tyrosine metabolism (Figures 5D, E). Among them, the signal pathways related to nervous system development are the AMPK signaling pathway and PI3K-Akt signaling pathway (Waite and Eickholt, 2010; Kong et al., 2020; Dai et al., 2021; Zhang et al., 2023), and more importantly, these two signal pathways have been proven to regulate the process of mitochondrial apoptosis (Zhao and Klionsky, 2011; Gao et al., 2020; Zheng et al., 2021; Gu et al., 2022).

Consequently, we investigated whether the *Mex3c* KO mice exhibited alterations in the AMPK signaling pathway. In hippocampus, while total AMPK (tAMPK) levels remained unchanged between WT and KO groups, phosphorylated AMPK (pAMPK) expression was significantly downregulated in *Mex3c* KO mice compared to WT controls (Figure 5F). We also examined the expression of SIRT1, a key downstream target of AMPK. *Mex3c* KO mice showed a marked reduction in SIRT1 protein levels. Furthermore, to assess the activation of PGC1a — a transcriptional coactivator regulated by SIRT1 — we measured its protein expression in both groups. The results showed a notable decrease in PGC1a activity in the KO group (Figure 5F). This aligns with previous findings indicating that SIRT1 positively regulates PGC1a expression through deacetylation mechanisms. Together, these results suggest that deletion of the *Mex3c* gene disrupts the AMPK/SIRT1/PGC1a signaling pathway, contributing to mitochondrial dysfunction and impaired ATP homeostasis in the brain. These alterations may ultimately underlie the development of ASD-like phenotypes observed in *Mex3c* KO mice.

## 4 Discussion

In the present study, we explored the effects of *Mex3c* gene deficiency on neurodevelopmental and behavioral phenotypes in mice. Our findings demonstrate that *Mex3c* KO mice exhibit pronounced social deficits and cognitive impairments, accompanied by neuronal injury, synaptic alterations, and mitochondrial dysfunction.

Behavioral analyses revealed that *Mex3c* KO mice displayed reduced social preference and impaired recognition of novel social partners in the three-chamber social interaction test. These features are consistent with autistic-like behaviors, which are characterized by social communication deficits and restricted social interaction. These findings are consistent with observations in several established ASD mouse models, such as *Shank3* and *Cntnap2* knockouts, which also display deficits in social interaction and communication (Bozdagi et al., 2010; Peñagarikano et al., 2011). Notably, these changes in this study emerged between postnatal day P30 and P50. However, since we did not assess earlier or later developmental stages, we cannot definitively determine the precise onset or progression of these changes. This consideration is important, as other studies have demonstrated that certain behavioral phenotypes in ASD models could emerge or intensify during specific developmental windows. For instance, *Shank3* Δex4–22 mice exhibit more pronounced social deficits and repetitive behaviors in adulthood compared to juvenile stages, indicating an age-related exacerbation of behavioral impairments (Kshetri et al., 2024). These findings underscore the importance of considering developmental timing when assessing behavioral phenotypes in ASD models. While *Mex3c* is not currently listed in the SFARI gene database, our data suggest that *Mex3c* deficiency may indirectly contribute to ASD-like phenotypes, potentially through AMPK/SIRT1/PGC1a pathways affecting neuronal development and energy metabolism, which have been implicated in ASD pathogenesis.

Beyond social deficits, *Mex3c* KO mice exhibited significant impairments in recognition memory and learning flexibility, as shown by the new object recognition and shuttle box tests. Our findings are consistent with previous studies indicating that *Mex3c* is highly expressed in the brain and contributes to cognitive processes. Previous study demonstrated that *Mex3c* deletion impairs cognitive function in mice via inhibition of autophagy, leading to synaptic dysfunction and neuronal loss (Wang et al., 2023). Previous research has also shown that *Mex3c* participates in neural development, energy metabolism, and immune regulation, providing a plausible biological basis for its involvement in neurodevelopmental disorders (Kuniyoshi et al., 2014; Du et al., 2020). At the neuronal level, we observed decreased NeuN expression in the hippocampus of *Mex3c* KO mice, suggesting compromised neuronal integrity. Morphological analyses further revealed reduced dendritic spine density on pyramidal neurons. This is in line with earlier findings that reported decreased expression of synaptic proteins PSD-95 and GAP-43 in *Mex3c* KO mice, which are essential for maintaining synaptic structure and function (Wang et al., 2023). These synaptic abnormalities may underlie the observed behavioral deficits, as impaired spine maturation is a hallmark of neurodevelopmental disorders, including ASD.

Mitochondrial analyses demonstrated decreased mitochondrial membrane potential in *Mex3c* KO mice. In addition, Transmission electron microscopy of hippocampal neurons in *Mex3c* KO mice revealed severe mitochondrial damage, including membrane blurring, crest loss, swelling, vacuolation, and overall structural deformation. These morphological abnormalities were accompanied by declines in mitochondrial membrane potential, respiratory chain complex activity, and ATP synthesis, implicating mitochondrial dysfunction as a driver of neuronal energy deficiency. Mitochondrial abnormalities have been widely reported in ASD, with prior studies identifying deficits in oxidative phosphorylation and ATP production (Rossignol and Frye, 2012; Trinchese et al., 2022; Frye et al., 2024). Our findings are consistent with these observations and further suggest that *Mex3c* deletion compromises mitochondrial function at both structural and functional levels.

Mechanistically, proteomic and transcriptomic analyses revealed that *Mex3c* deletion disrupted multiple cellular pathways, with notable downregulation of the AMPK/SIRT1/PGC1a axis. This pathway is a central regulator of cellular energy homeostasis and mitochondrial biogenesis (Cantó and Auwerx, 2009; Garcia and Shaw, 2017; Jia et al., 2023). *Mex3c* KO mice exhibited reduced expression of p-AMPK, AMPK, SIRT1, and PGC1a in hippocampal neurons. AMPK maintains cellular energy balance by regulating NAD+ levels, which activates SIRT1, promoting PGC1a-mediated mitochondrial biogenesis and maintaining mitochondrial membrane integrity (Cantó et al., 2009; Rakshe et al., 2024). Disruption of this pathway likely underlies the mitochondrial impairments observed in our model.

While our study provides compelling evidence linking *Mex3c* deficiency to ASD-relevant phenotypes, it is important to acknowledge several limitations. As previous mentioned, *Mex3c* is not currently listed in the SFARI gene database of ASD-associated genes. However, emerging research has linked *Mex3c* to critical biological processes relevant to neurodevelopment, including antiviral immune responses (Kuniyoshi et al., 2014), metabolic regulation (Jiao et al., 2012; Chao et al., 2019), and autophagy inhibition (Wang et al., 2023). These findings, together with our current data, support the hypothesis that *Mex3c* may influence ASD-related pathways through converging mechanisms involving metabolism, neuroinflammation, and mitochondrial function.

Future research should focus on genetic association studies to determine whether *Mex3c* variants confer ASD risk in human populations. Genome-wide association studies (GWAS) and gene network analyses will be crucial to further define the contribution of *Mex3c* to ASD susceptibility. Additionally, in-depth mechanistic studies are warranted to elucidate downstream effectors of the AMPK/SIRT1/PGC1a pathway in *Mex3c* deficiency and explore the therapeutic potential of pathway agonists in ASD models.

Collectively, our findings suggest that *Mex3c* deficiency induces age-dependent neuronal and behavioral deficits in mice, potentially through impaired synaptic plasticity and mitochondrial dysfunction. Although the direct involvement of *Mex3c* in ASD remains to be confirmed, our study provides new insights into the functional role of *Mex3c* in brain development and behavior, highlighting it as a candidate gene for further investigation in neurodevelopmental disorders.

## Data Availability

The datasets presented in this study can be found in online repositories. The names of the repository/repositories and accession number(s) can be found in the article/Supplementary material.
