# Supplementary material for: Deletion of Mex3c gene leads to autistic-like behavior in mice by inhibiting AMPK signal pathway
Source: Front Behav Neurosci. 2025 May 20;19:1551440. doi: 10.3389/fnbeh.2025.1551440 (PMC12129987; doi:10.3389/fnbeh.2025.1551440)

**Table S1. Summary of behavioral, histological, and molecular quantifications in WT and Mex3c KO mice**

| Experiemtns | Parameters | WT (mean±SD) | KO (mean±SD) | P |
| --- | --- | --- | --- | --- |
| **Open Field Test** | Time in center (%) | 18.74 ± 2.70 | 9.82 ± 1.57 | 3.96 × 10⁻¹² |
|  | Time in center (s) | 77.57 ± 9.77 | 51.78 ± 8.41 | 1.21 × 10⁻⁸ |
|  | Total distance traveled (cm) | 72.46 ± 8.23 | 63.74 ± 12.41 | 0.1435 |
| **Elevated Plus Maze** | Open arm residence time (OT, %) | 24.97 ± 7.39 | 11.75 ± 1.85 | 1.90 × 10⁻⁷ |
|  | Entries into open arm (OE, %) | 65.09 ± 5.11 | 29.35 ± 2.97 | 7.78 × 10⁻²¹ |
| **Three-Chamber Social Test** | WT: Stranger 1 vs. empty (s) | 28.94 ± 3.68 | 6.06 ± 2.59 | 4.82 × 10⁻⁷ |
|  | KO: Stranger 1 vs. empty (s) | 15.34 ± 3.14 | 16.96 ± 4.41 | 0.519 |
|  | WT vs. KO (Stranger 1 time) | 28.94 ± 3.68 | 15.34 ± 3.14 | 9.08 × 10⁻⁵ |
| **Nissl Staining** | CA1 region | 79.97 ± 1.41 | 66.80 ± 1.53 | 8.55 × 10⁻⁴ |
|  | CA3 region | 82.30 ± 2.85 | 59.87 ± 1.19 | 5.05 × 10⁻⁴ |
|  | DG region | 90.97 ± 2.17 | 73.33 ± 2.25 | 1.35 × 10⁻³ |
| **IHC (NeuN-positive cells)** | CA1 region | 35.53 ± 2.39 | 27.72 ± 1.92 | 2.26 × 10⁻² |
|  | CA3 region | 34.14 ± 1.93 | 29.33 ± 1.50 | 4.97 × 10⁻² |
|  | DG region | 35.82 ± 1.99 | 27.17 ± 1.69 | 9.43 × 10⁻³ |
| **TEM (Mitochondrial damage)** | % of damaged mitochondria | 9.50 ± 0.80 | 56.73 ± 6.13 | 4.15 × 10⁻⁴ |
| **Golgi-Cox Staining** | Dendritic spine density (hippocampus) | 1.36 ± 0.07 | 0.72 ± 0.04 | 2.72 × 10⁻⁴ |
|  | Dendritic spine density (cortex) | 1.16 ± 0.11 | 0.60 ± 0.03 | 2.29 × 10⁻³ |
| **JC-1 Assay (MMP)** | PND50 | 91.28 ± 0.61 | 78.63 ± 1.09 | 6.64 × 10⁻¹⁰ |
|  | PND30 | 94.85 ± 0.95 | 91.45 ± 0.17 | 1.35 × 10⁻⁵ |
| **ATP Assay** | PND50 | 193.81 ± 3.70 | 157.94 ± 8.04 | 3.89 × 10⁻⁶ |
|  | PND30 | 561.72 ± 18.79 | 400.39 ± 10.67 | 1.24 × 10⁻⁸ |
| **Western Blot Quantification** | tAMPK/GAPDH | 1.00 ± 0.00 | 1.15 ± 0.19 | 0.332 |
|  | pAMPK/tAMPK | 1.00 ± 0.00 | 0.31 ± 0.04 | 1.32 × 10⁻⁵ |
|  | SIRT1/GAPDH | 1.00 ± 0.00 | 0.26 ± 0.03 | 3.49 × 10⁻⁶ |
|  | PGC-1a/GAPDH | 1.00 ± 0.00 | 0.57 ± 0.09 | 2.04 × 10⁻³ |
|  | GAP-43/GAPDH | 1.00 ± 0.00 | 0.10 ± 0.01 | 4.05 × 10⁻⁸ |
|  | PSD-95/GAPDH | 1.00 ± 0.00 | 0.53 ± 0.05 | 1.46 × 10⁻⁴ |

Western Blot

tAMPK


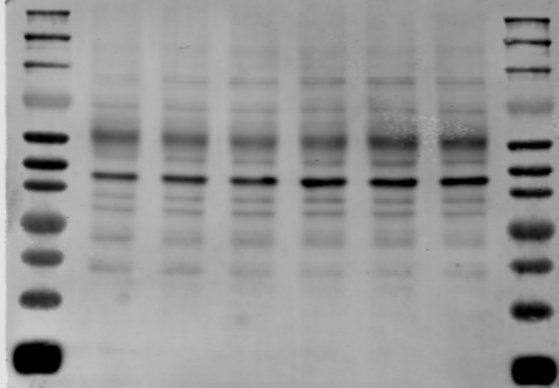


pAMPK


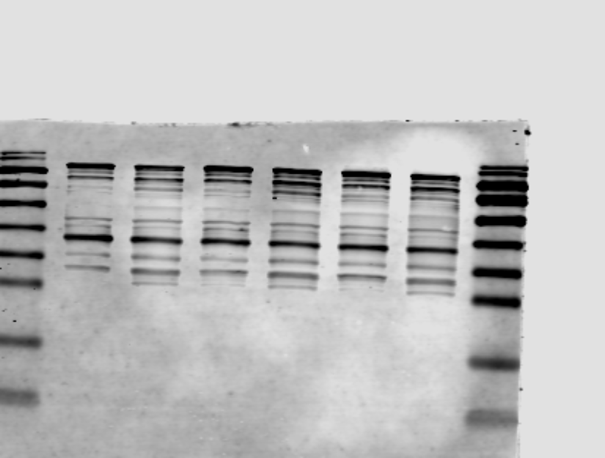


SIRT1


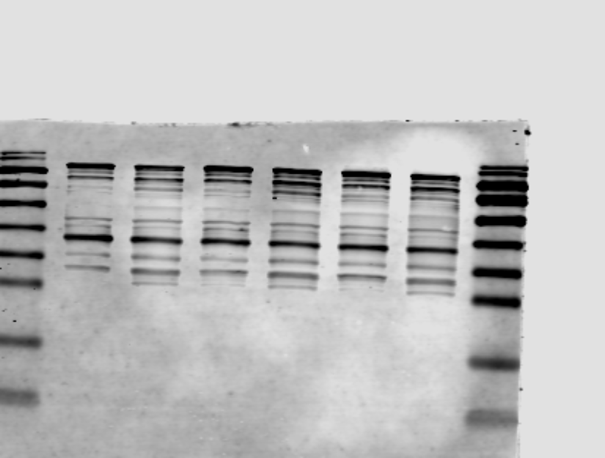


PCG1a


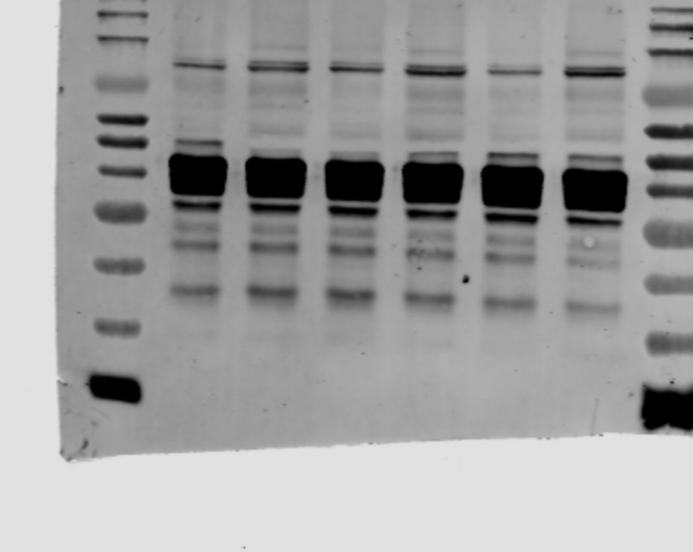


GAPDH (PCG1a)


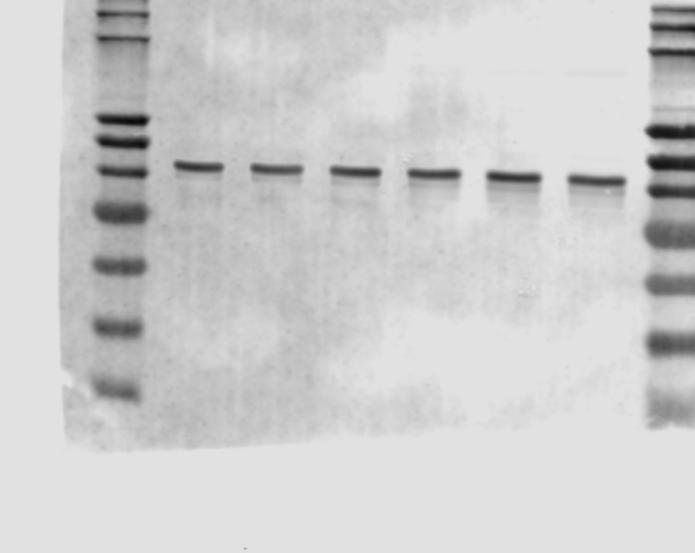


PSD95


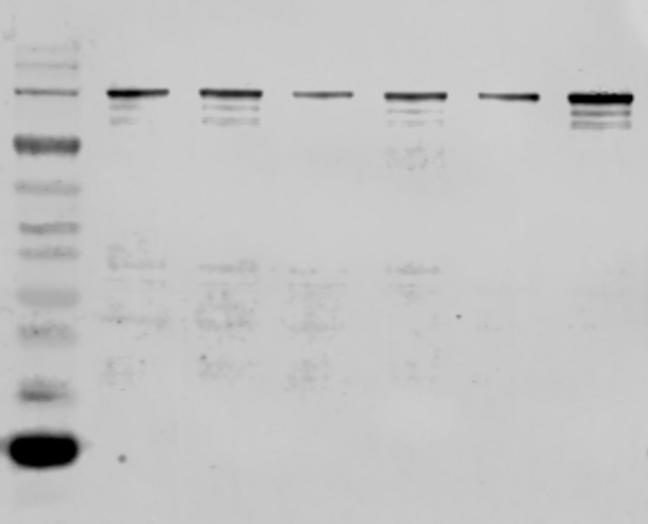


PSD95(GAPDH)


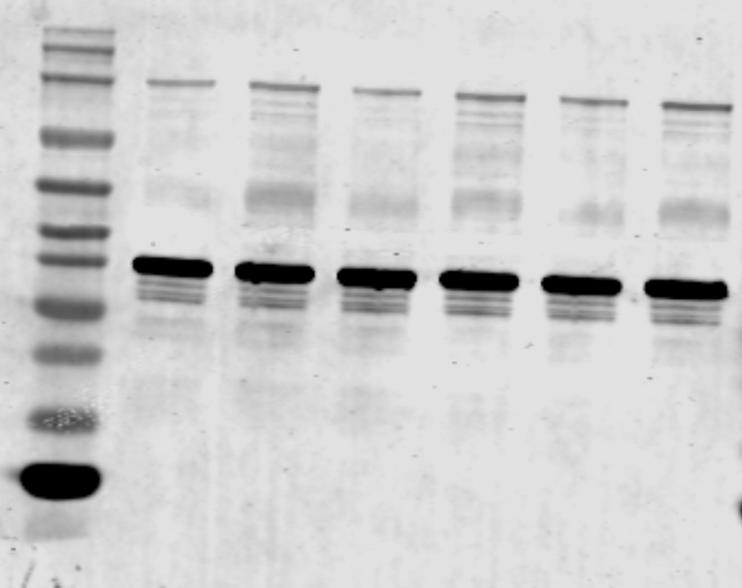


GAP43


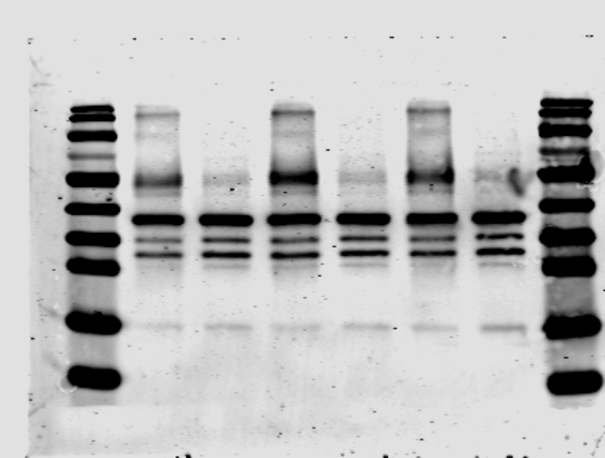


Golgi-COX staining (Bregma −1.79mm）

Cortex

Hippocampus


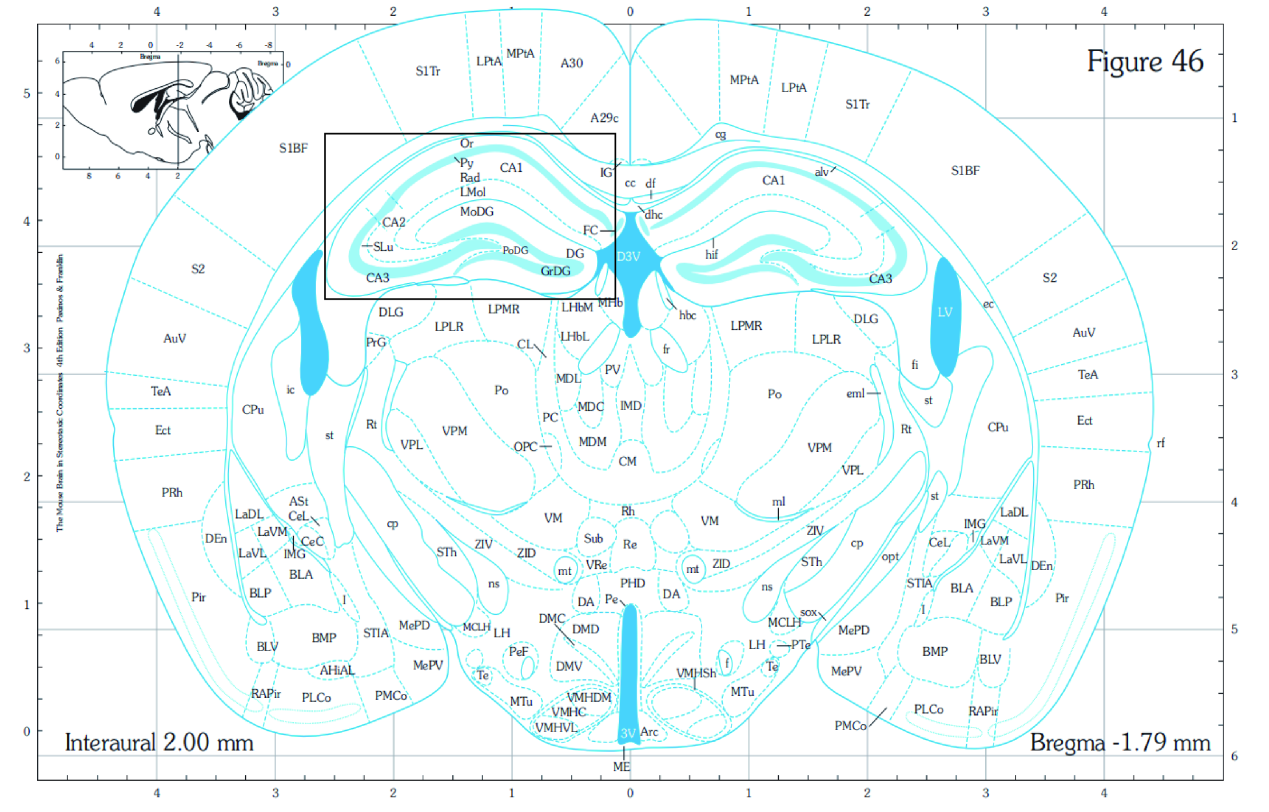


Nissl staining (Bregma −1.67mm）


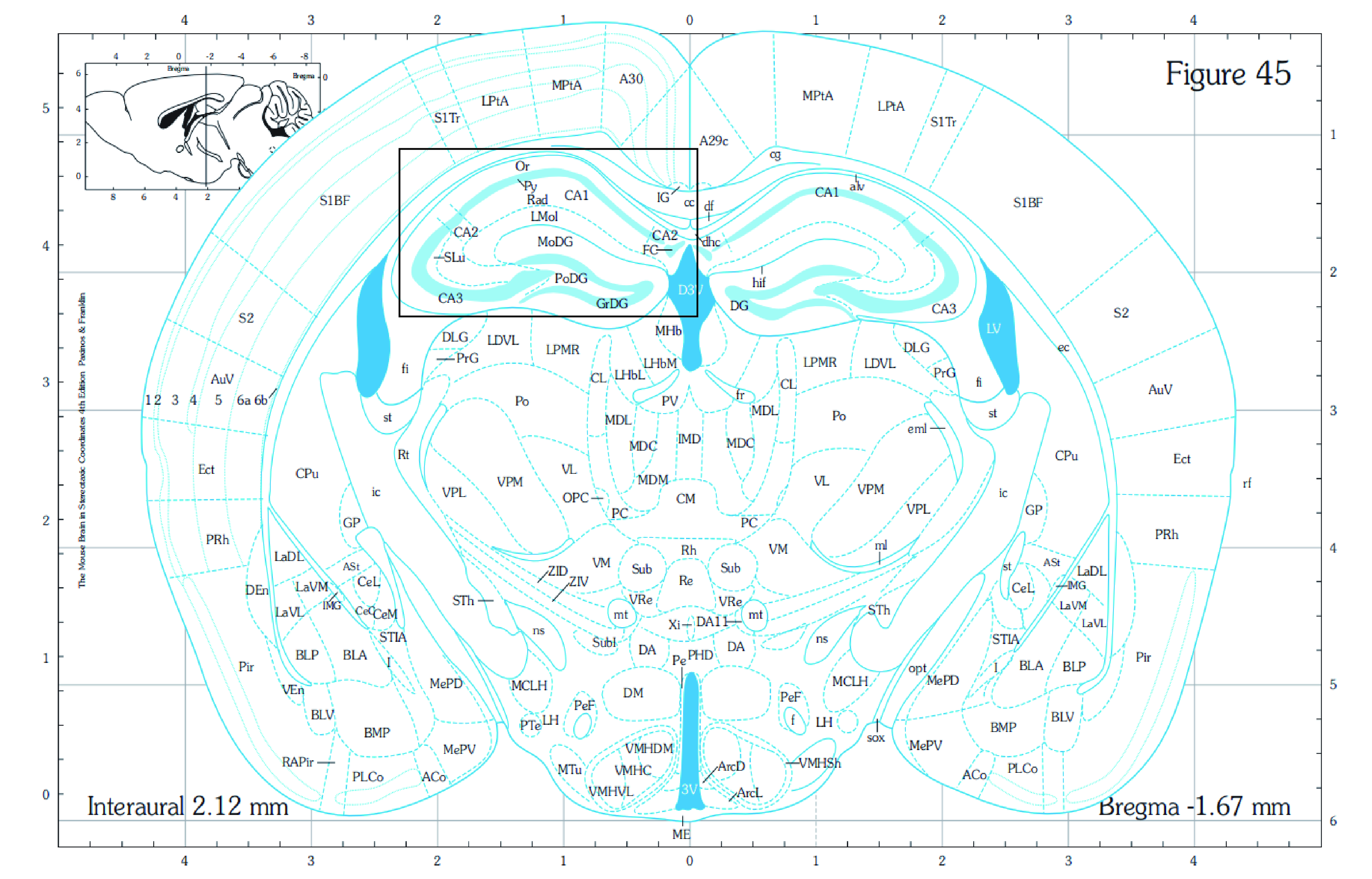


NeuN immunohistochemistry (Bregma −1.67mm）


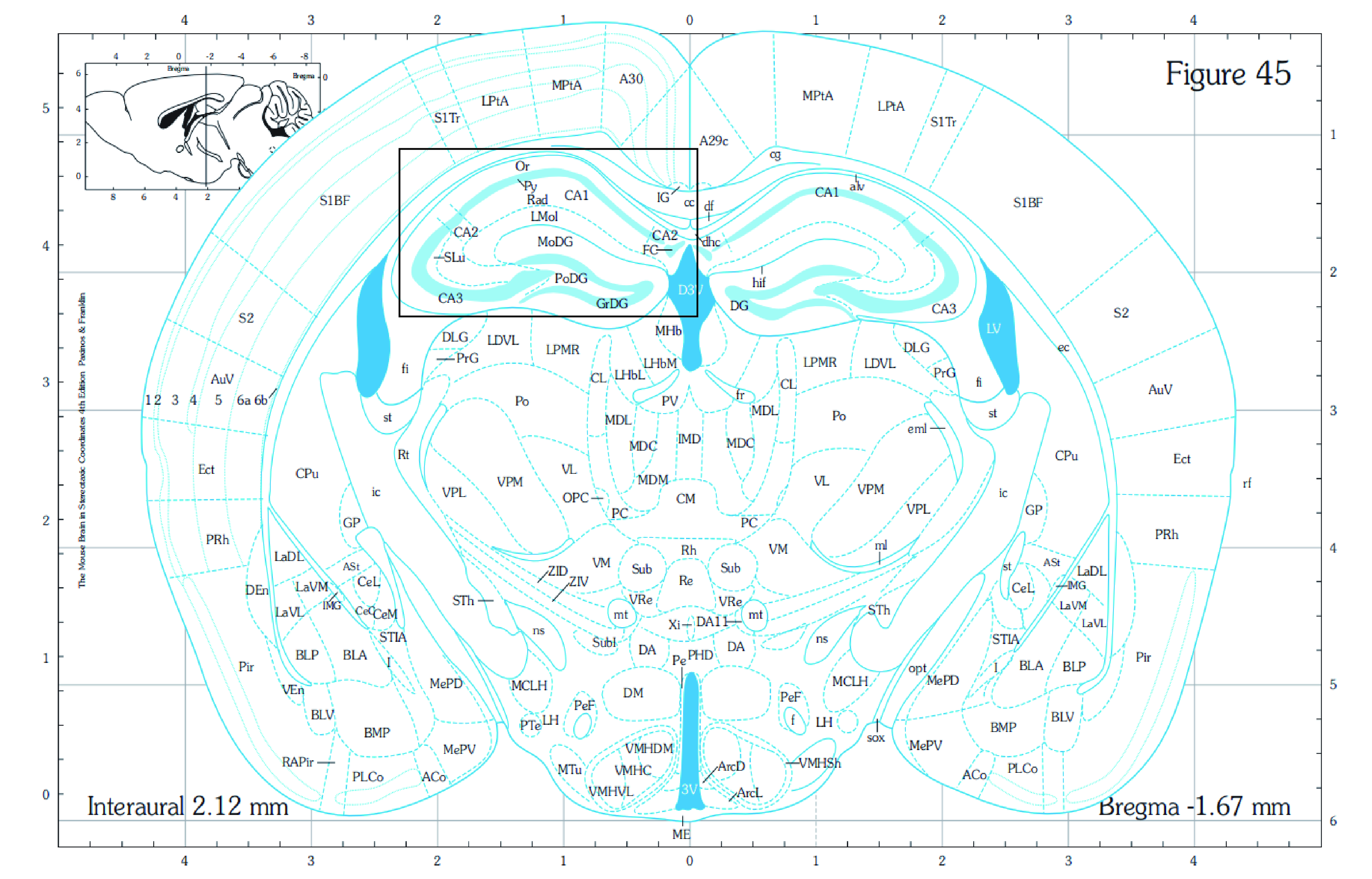

Supplement: Supplementary file 1 [file Table_1.docx]
